# A catheterizable serous-lined urinary outlet associated with the ileal bladder augmentation Abol-Enein and Ghoneim procedure: a safe and reliable procedure in children

**DOI:** 10.3389/fped.2024.1273505

**Published:** 2024-02-29

**Authors:** Hortense Alliot, Toussaint Tapsoba, Annabel Paye-Jaouen, Yaqoub Ashkanani, Eliane Josset-Raffet, Lise Natio, Matthieu Peycelon, Alaa El-Ghoneimi

**Affiliations:** Department of Pediatric Surgery and Urology, National Reference Center for Rare Urinary Tract Malformations (MARVU), University Hospital Robert-Debre, APHP, Université Paris Cité, Paris, France

**Keywords:** bladder exstrophy–epispadias complex, urinary reservoirs, continent diversion, urinary catheterization, urologic surgical procedures, surgical stomas, child, age

## Abstract

**Purpose:**

This study aims to evaluate the long-term outcome of the serous-lined extramural continent catheterizable outlet procedure (SLECCOP) associated with ileal bladder augmentation in children.

**Methods:**

This was a monocentric and retrospective study (2002–2021) that included children (<18 years) undergoing the SLECCOP associated with W-shaped ileocystoplasty with a catheterizable channel (Abol-Enein and Ghoneim procedure). Patients who received other types of bladder augmentation or W-shaped ileocystoplasty without a catheterizable channel were excluded. Patient records were reviewed for demographic information, surgical data, and long-term outcomes.

**Results:**

This study included 52 children [33 boys, median age: 8.5 (0.8–18) years]. Pathological conditions included 28 children with the bladder exstrophy and epispadias complex (BEEC), 11 with neurogenic bladders, and 13 with other pathologies. Two patients underwent total bladder substitution. Thirty-four (65%) patients had bladder neck reconstruction (BNR), with 23 undergoing the SLECCOP and ileocystoplasty and 11 having prior BNR. All stomas, except for two, were umbilical, and were associated with omphaloplasty in 28 patients with the BEEC. A total of 40 stomas were created using the appendix (77%) and 12 with a Monti tube (23%). Stoma-related complications included cutaneous strictures (*n *= 2, 4%) and leaks (*n *= 10, 19%), all treated by dextranomer/hyaluronic acid copolymer injection (*n *= 10). A redo surgery was required in three patients: extraserosal wrapping was performed for persistent leakage (*n *= 2, 4%), and surgical revision was required for the Monti tube procedure (*n *= 1, 2%). Three patients (6%) underwent dilatation for transient stoma stenosis. Leakage occurred in 20% of appendix channels (*n *= 8/40) and 17% of Monti tubes (*n *= 2/12). Strictures were reported in 3% of appendix channels (*n *= 1/40) and 8% of Monti tubes (*n *= 1/12). Bladder stones developed in four patients (8%). Channel leakage persisted in one patient (2%) at a median follow-up of 4.4 years (IQR 1.4–9.7).

**Conclusion:**

W-Ileal bladder augmentation with the SLECCOP is an efficient technique for treating children with incontinence caused by different etiologies. The rate of channel complication is very low, specifically for strictures, in this complex population of patients.

## Introduction

Lower urinary tract reconstruction remains a challenging task for surgeons to achieve continence in children while preserving the upper tract. Although many new procedures have been attempted, indications for external continent diversion are continuously being updated, especially in complex urogenital anomalies such as the bladder exstrophy–epispadias complex (1–3). Since the first description of the Mitrofanoff principle (4), the choice of conduit remains a topic of debate regarding their type, site, and the method of integration into an augmented bladder (5–8). These procedures are debated mainly because they tend to produce inconsistent long-term results. The serous-lined extramural tunnel technique, as described by Abol-Enein and Ghoneim, has been proven effective in adults and recently in children (9–12). Therefore, our objective in this study is to retrospectively evaluate this technique. We have studied our experience of using this procedure in a cohort of children with complex lower urinary tract reconstruction who needed bladder augmentation (BA) and external continent diversion. Our primary outcomes focused on stoma complications. Urethral leakage and bladder stone development were also explored for each patient.

## Materials and methods

We retrospectively studied all patients who underwent a continent catheterizable stoma procedure using the serous-lined extramural technique associated with W-ileal bladder augmentation at the Department of Pediatric Urology of Robert Debré University Hospital in Paris, France, between September 2002 and October 2021. Data on age, sex, initial pathology, indications, therapeutic details, and follow-up results were collected.

All patients and their parents received detailed information on the indications and the modalities of follow-up, specifically on clean intermittent catheterization (CIC) and its lifelong necessity. They participated in education sessions with stoma therapists, and we let them choose the cutaneous site for the urinary diversion. A specialized pediatric psychologist from the department also participated in these sessions and evaluated their acceptance for the modification of their body images and for an understanding of the constraints posed by the CIC procedure.

### Operative details

#### Ileocystoplasty

The bladder approach was performed extraperitoneally through a transverse Pfannenstiel incision (for those with a neurogenic bladder and others) or a median suprapubic incision (for bladder exstrophy). The bladder was dissected from the peritoneum. A midline incision of the bladder was extended anteriorly to the bladder neck (BN) and posteriorly to 2 cm from the trigone (bivalved bladder). In the case of bladder neck reconstruction (BNR), a bladder neck surgery was performed first and the ileal segment was then sutured directly to the anterior edges of the bladder neck. The ureters were reimplanted, when needed, preferentially in the bladder segment or otherwise in the left side of the W-shaped segment in an extramural type. When possible, an opening was made through the peritoneum to allow extraperitonization of the bladder segment at the end of the surgery. The ileal segment was isolated 25 cm proximal to the ileocecal junction. The segment measured 20 cm on average. The technique entailed creating a serous-lined extramural trough within a folded, isolated ileal segment, into which the appendix or the Monti reconfigured ileal tube was implanted. The segment was reconfigured as W-shaped; the antimesenteric border was opened all along the tube, and the detubularized edges were sutured together by a continuous 4/0 polyglactin suture, leaving the right limb of the W open to serve as the serous-lined trough.

#### Preparation of the diversion conduit

**Figure 1:**
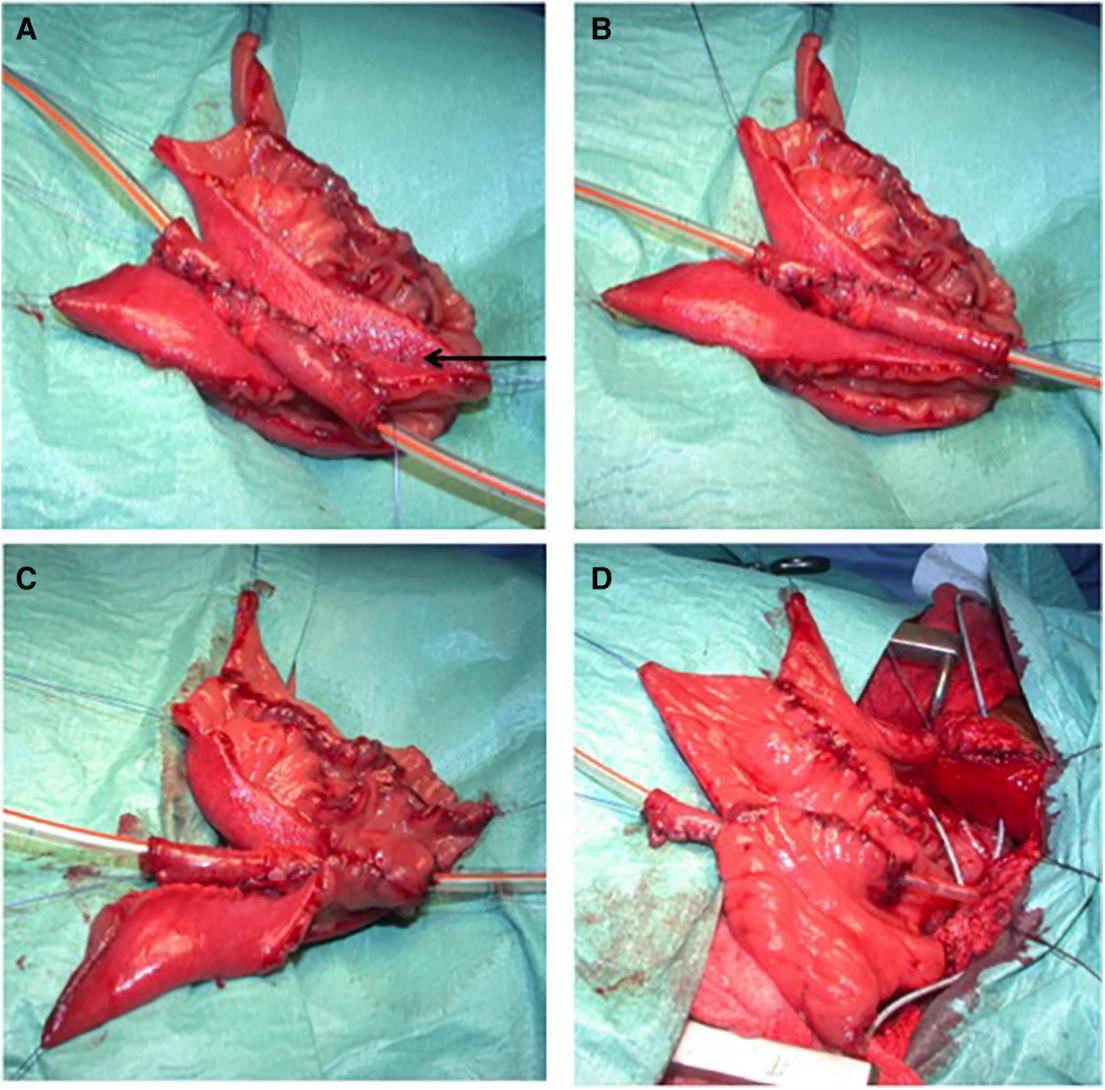
The construction of an extramural trough. (**A**) Two parallel lines of 4 cm of length are designed by diathermy on the serosa. The two adjacent limbs of the trough are approximated (arrow) using a 4/0 monofilament absorbable polydioxanone seromuscular suture passing through the mesenteric windows if needed. (**B**) The appendix, or the Monti segment (as in this picture), is inlaid in its corresponding serous-lined trough. (**C**) The trough is designed to fit the appendix or Monti segment in a tight pattern between the seromuscular sutures and the free edges of the detubularized segment. An anastomosis is done between the spatulated appendix or the Monti segment and the detubularized segment. (**D**) The final aspect of the extramural trough.

**Figure 2:**
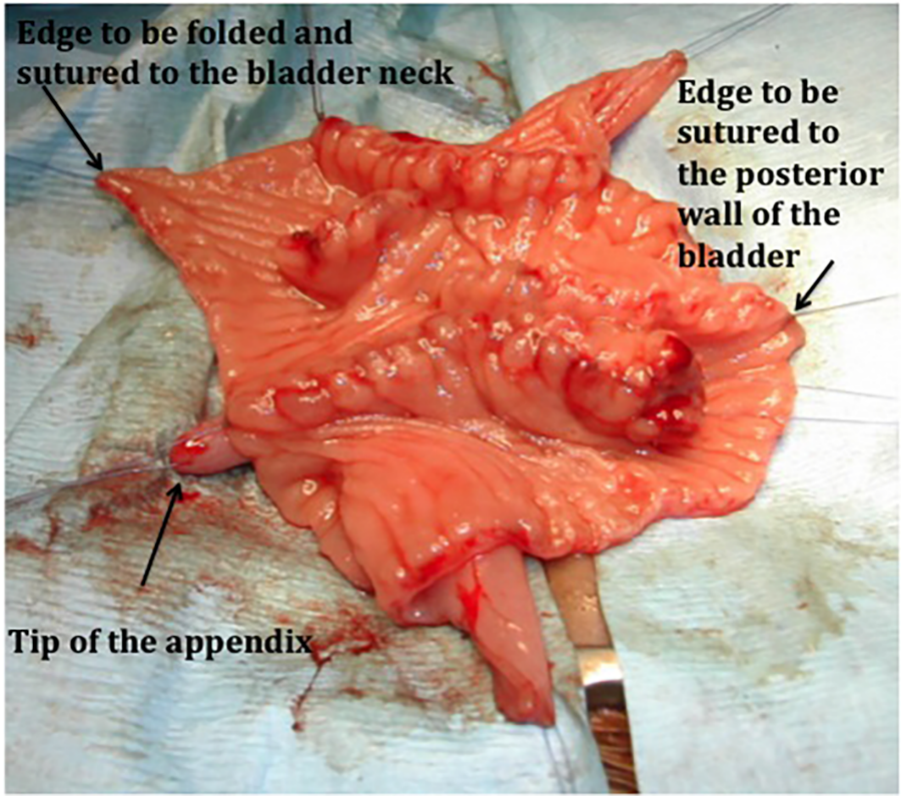
A suture of the ileal augment to the bladder and skin stoma in a bladder exstrophy. The detubularized reconfigured W segment is sutured to the bladder wall. Note that the free edge to be folded to the bladder neck has no anastomosis to give the optimal condition for healing.

**Figure 3:**
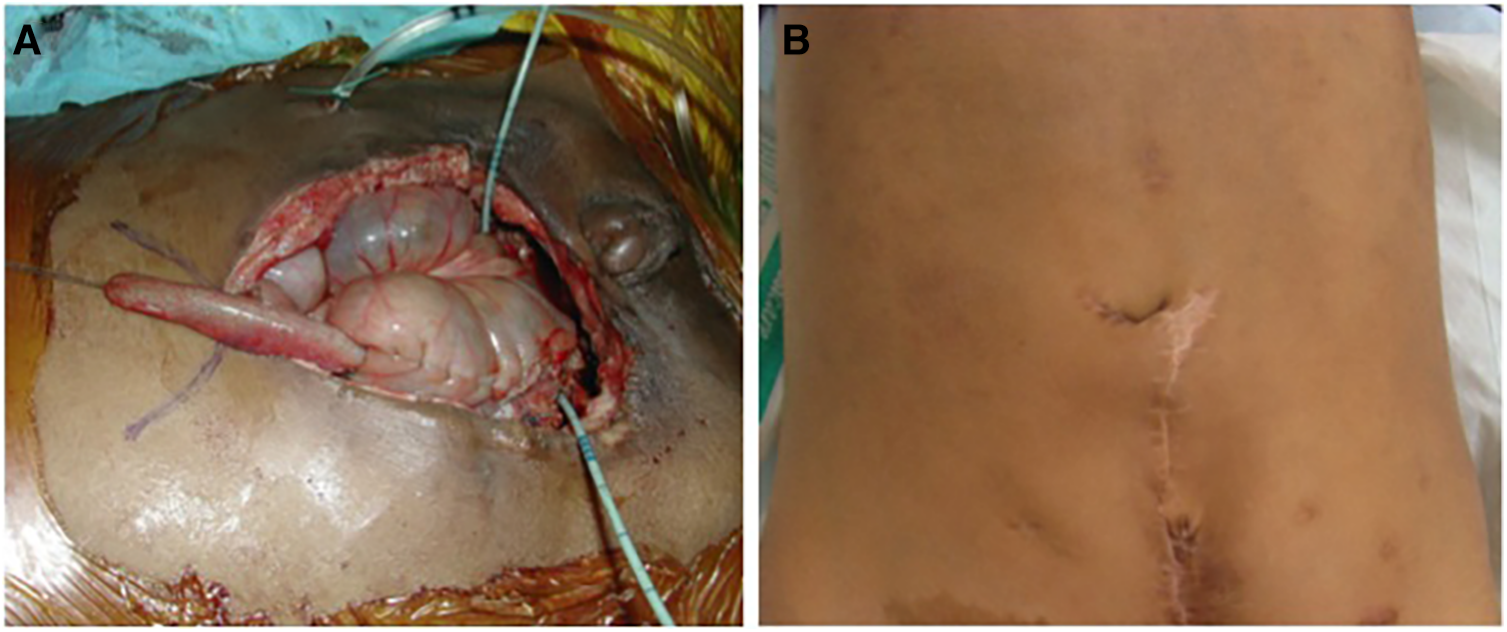
The final aspect of the same case as in Figure 2. (**A**) The appendix is sutured to the new umbilicus skin flap. Note that the appendix is easily pulled to this position. (**B**) The external aspect 6 months after surgery.

When the appendix was available, it was disconnected from the base of the cecum, with the base of the appendix remaining attached to the cecum. The appendicular mesentery was dissected at minimum to allow mobilization of the appendix. On whether the proximal or the distal part of the appendix would be sutured to the ileal segment, a decision was taken based on the anatomical possibility. If the appendix was not available, a short ileal segment of 2 cm was prepared according to Monti's principle. The extramural trough was prepared as shown in Figure 1. The appendix was sutured to the skin, with our preference being mostly at the umbilicus, only at the end of the surgery. In the case of bladder exstrophy, a neo-umbilicus was created and sutured to the spatulated appendix (Figures 2, 3). The conduit was drained by using a Ch12 catheter for 15 days, after which the suprapubic catheter was closed, and CIC was performed through the conduit. The interval between CIC sessions was progressively increased; the continuous night drainage was usually discontinued after 3 weeks. The process of irrigating the augmented bladder started 48 h after the surgery, with irrigation performed daily using clean water. In the case of bladder substitution, ureters were implanted in a gun-barrel fashion on the other arm of the “W” using the same technique.

### Postoperative follow-up criteria

The follow-up was provided by the surgeon, specialized nurses, and pediatric nephrologists. Bladder irrigation was performed by the patients or their families once per day with clean water (60–100 ml) during the first year, with the frequency adjusted according to the amount of mucus. In general, the patients were kept on regular irrigation as far as possible. During the first year, the children were evaluated by the surgical team every 3 months, then every 6 months for the subsequent year, and then annually after that. At each visit, a detailed calendar of CIC and its modalities was revised by the outpatient nurse. The children were considered continent if they remained dry for at least 3 h without catheterization. Any leakage from the urethra at shorter intervals or during the night was considered incontinence*.* Complications of the stoma were considered significant if they required any specific measure. Stenosis was considered if any redo surgery was needed. When a dilatation of the channel under general anesthesia was required to unravel the stenosis, we considered it transient stenosis. Any leakage from the stoma was considered a failure and needed a full urodynamic study evaluation; a redo surgery was attempted initially by endoscopic injection, and if this resulted in failure, open surgery was performed. Only febrile urinary tract infections (UTIs) were reported, while cystitis cases were not included. Therefor, we considered each UTI an early complication if it occurred within 30 days postoperatively.

### Statistical analysis

Statistical analyses were performed using Numbers v11.2 and RStudio v.1.3.1093. Descriptive analyses and comparative univariate analyses (Fisher's exact test) were conducted to compare stoma complications rates between different population groups.

## Results

### Demographic results

A total of 52 patients (33 boys and 19 girls) who were operated at a median age of 8.5 years (0.8–18) were included. A flowchart of this study is presented in Table 1.

**Table 1:** Flowchart.

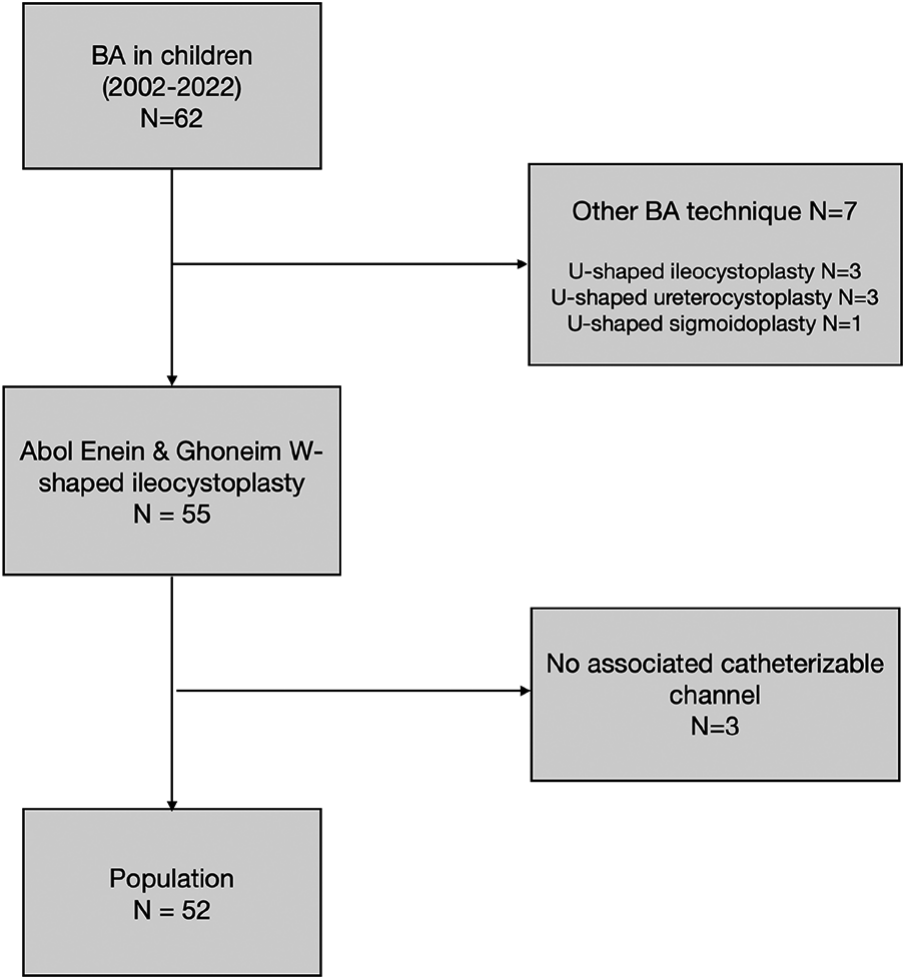

#### Etiology

Table 2 presents the etiologies observed in the study cohort, which are listed as follows: bladder exstrophy and epispadias complex (BEEC) (*n* = 28, 53.8%), including two cloacal exstrophy and two incontinent epispadias cases; neurogenic bladder (*n* = 11, 21.2%); bilateral ectopic ureters (*n* = 2, 3.8%); urogenital sinus (*n* = 2, 3.8%); one case of posterior urethral valve; one case of vesicoprostatic rhabdomyosarcoma; one case of bladder fibrosis after refractory hemorrhagic cystitis; one case of prune belly syndrome; one case of bladder agenesis associated with an omphalocele; one case of severe dyssynergia female hypospadias; one case of obstructive megaureter on a single kidney; one case of Cornelia de Lange syndrome with major bladder neck insufficiency and sphincter deficiency; and one case of complex uropathy bilateral kidney dysplasia. Two patients underwent total bladder substitution with an ileal pouch: one with vesicoprostatic rhabdomyosarcoma who underwent radical cystectomy and another with bladder agenesis associated with an omphalocele.

**Table 2:** Type of initial pathology.

Initial pathology	*N*	%
Classic BEEC	24	46.2
Cloacal BE	2	3.8
Incontinent epispadias	2	3.8
Neurogenic bladder	11	21.2
Bilateral ectopic ureters	2	3.8
Urogenital sinus	2	3.8
Complex uropathy with bilateral kidney dysplasia	1	1.9
PUV	1	1.9
Vesicoprostatic rhabdomyosarcoma	1	1.9
Bladder fibrosis after refractory hemorrhagic cystitis	1	1.9
Prune belly syndrome	1	1.9
Bladder agenesis	1	1.9
Female hypospadias	1	1.9
Obstructive megaureter on a single kidney	1	1.9
Cornelia de Lange syndrome	1	1.9

#### Previous surgery

Only 13 patients (25%) had never undergone bladder surgery previously. Among them, 69.3% (9/13) had a neurogenic bladder.

The most frequent surgery performed before the serous-lined extramural continent catheterizable outlet procedure (SLECCOP) was bladder exstrophy closure in 25 out of 52 patients (48.1%) (24 cases of classic bladder exstrophy and 1 case of cloacal exstrophy). Previous BNR was performed in 11 out of 52 patients (21.2%), either alone or associated with the SLECCOP procedure in 23 out of 52 patients (44.2%).

In all cases of patients (*n* = 52), there was an association with a continent catheterizable channel , with the appendix being the continent urinary diversion conduit in 40 patients (76.9%) and the ileal Monti tube in 12 patients (23.1%). The skin site of the diversions was the umbilicus for all patients, except for two whose stoma was in the right iliac fossa. Omphaloplasty was associated with all BEEC patients (*n* = 28/52). The other associated surgeries included the following: bladder neck closure (*n* = 6, 11.5%), bladder neck dextranomer/hyaluronic acid copolymer (Dx/HA) injection (*n* = 5, 9.6%), bladder neck suspension (*n* = 1, 1.9%), and vesicoureteral reimplantation (*n* = 19, 36.5%).

The surgical management and subsequent complications of each patient are reported in Tables 3 and 4, respectively.

**Table 3:** Surgical management of urethral leakage and results.

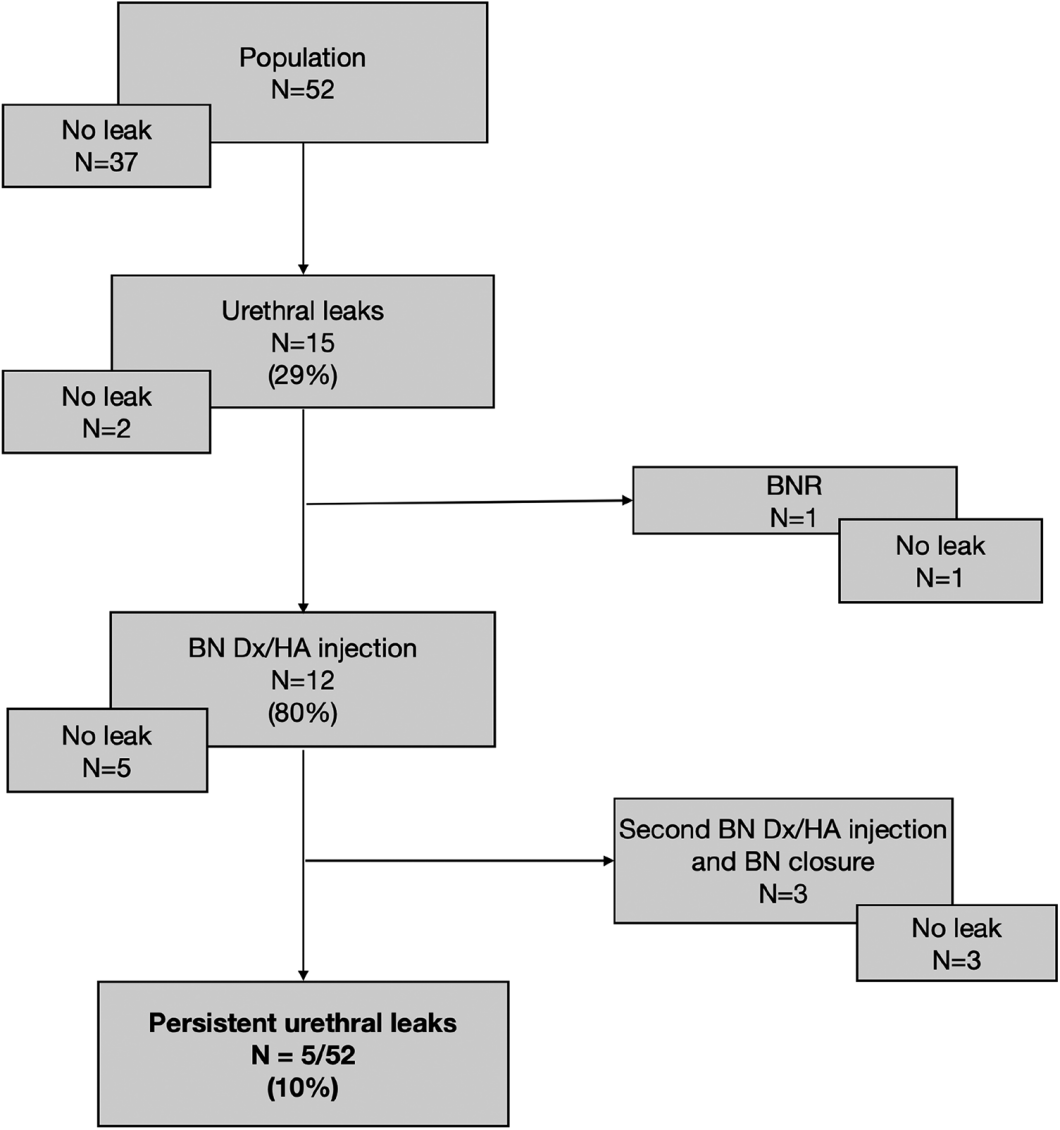

**Table 4:** Surgical management of stoma complications and results.

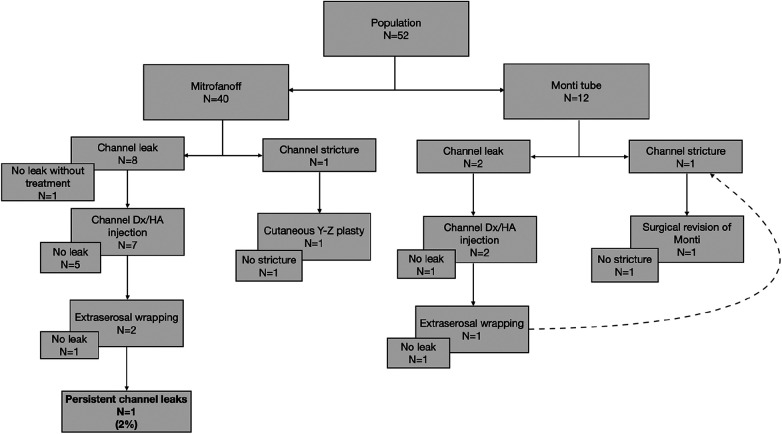

### Primary outcomes

#### Stoma outcomes

Both patients with stoma strictures (*n* = 2, 3.8%) underwent surgical treatment. One of the strictures needed a channel redo, whereas the other one was resolved with suprafascial revision using a cutaneous Y-Z plasty. No strictures remained in these patients, rendering the performance of CIC easy. Three patients (5.8%) underwent dilatation in consultation for transient stoma stenosis at the skin side. Channel leakage (*n* = 10, 19.2%) was treated by Dx/HA injection (*n* = 10), with successful results seen in seven patients. A redo surgery was required for three patients: extraserosal wrapping (*n* = 2, 3.8%) and surgical revision of Monti tubes (*n* = 1, 1.9%). Despite Dx/HA injection and extraserosal rewrapping, channel leakage persisted in one patient (1.9%) at a median follow-up of 4.4 years (IQR 1.4–9.7).

We did not highlight any factor impacting channel outcomes: neither the surgical technique nor the initial pathology influenced the frequency of leaks or strictures.

Leakage occurred in 20% of appendix channels (*n* = 8/40) and 16.7% of Monti tubes (*n* = 2/12) (*p* > 0.05). Strictures occurred in 2.5% of appendix channels (*n* = 1/40) and 8.3% of Monti tubes (*n* = 1/12) (*p* > 0.05).

Leakage occurred in 21.4% of channels in patients with the BEEC (*n* = 6/28) and in 18.2% in patients with a neurogenic bladder (*n* = 2/11) (*p* > 0.05). Strictures occurred in 7.1% of channels in patients with the BEEC (*n* = 2/28), whereas no channel strictures were reported in patients with a neurogenic bladder (*n* = 0/11) (*p* > 0.05).

### Secondary outcomes

#### Urethral leakage

In our population of complex uropathy, where BNR was associated with the SLECCOP procedure in almost half of the patients (*n* = 23/52, 44.2%), urethral leakage was observed in 15 out of 52 patients (28.8%). Twelve patients (*n* = 12/15, 80%) had primarily been treated with a BN injection of Dx/HA, and this treatment was successful in 5 out of 12 patients (41.6%), allowing them to stay dry for 3 h. Three patients (*n* = 3/12, 25%) needed another BN injection and then BN closure. Five patients in our population (*n* = 5/52, 9.6%) could not achieve the 3-h dryness interval despite BN injection. Concerning three patients who did not receive BN injection, urethral leakage resolved spontaneously in two, whereas the remaining one underwent BNR from the outset.

#### Bladder stones

Bladder stones developed in 4 out of 52 patients (7.7%). Each patient underwent an endoscopic procedure for bladder stone extraction; among them, one experienced recurrence and needed a secondary endoscopic procedure. Another one needed surgical cystotomy after endoscopic exploration to extract a bulky stone.

#### UTI

Early pyelonephritis occurred in 6 out of 52 patients (11.5%), and delayed pyelonephritis was observed in 6 other patients. None of these patients presented bladder stones. No patient reported life-threatening infections.

## Discussion

Bladder augmentation with continent diversion is a major surgical decision to make for children and their families, as it entails the performance of a lifetime CIC and medical follow-up. For this reason, other therapeutic measures should be attempted first, and bladder augmentation should be considered only in cases where all other alternatives have failed. The goals of bladder augmentation in children are mainly to achieve continence and to protect the upper urinary tract. For emptying the augmented bladder, CIC is required, which is ideally performed through the urethra, especially in patients with a neuropathic bladder. Our preference is urethra catheterization; however, unfortunately, the urethra is unsuitable for catheterization in most patients with bladder exstrophy after BNR and in some patients with a neuropathic bladder who exceptionally undergo repeated bladder neck surgeries or closures. Among these indications, the association of bladder augmentation and continent outlet in children is a frequent indication.

Since the first description of the Mitrofanoff principle (4), achieving a catheterizable continent channel remains a challenge. The concept of the serous-lined extramural valve technique of Abol-Enein and Ghoneim is derived from the unidirectional principle of antireflux ureteral reimplantation proposed by the same authors in 1994 (11, 13). The mechanism of this valve, as explained by the authors, relies on two integrated components to provide continence. The passive tubular resistance of the outlet prevents leakage at low pressure while resting, and the valve system incorporated within the reservoir wall provides a dynamic mechanism during reservoir filling and/or at high-pressure spikes (11).

After their first description in adult patients, the same institution had also published their experience in children, comprising a large cohort of 60 children in a period of 15 years (12). A total of 55 patients (91.6%) were included, with stoma-related complications being leaking stoma in 5 patients (8.4%), stomal stenosis in 6 (10%), parastomal hernia in 2 (3.3%), and reservoir stones in 8 (13.3%). The reoperation rate was 18.3% (11 patients). Soygur et al. reported no single complication in their series of 12 patients using the same procedure (14). Considering the different types of the most commonly practiced continent urinary diversions (the valve system described by Mitrofanoff, the nipple valve system, and the ileal plication called the “Indiana pouch”), Kaefer at al. found similar rates of continence, with the most important element to consider being the patient's anatomy (15). In recent studies, complication rates were found to vary, with urinary continence reported at 88%–97%, stoma stenosis at 13.7%, stoma revision at 13.7%, and persistent urinary leak at 3.7% (16, 17). Abdelhalim et al. compared the channel complication rates in 120 children who underwent the continent channel procedure using three different techniques (appendicovesicostomy, tapered ileum, or Monti ileal tubes). Associated bladder augmentation was performed in 68.3% of patients, and an ileal reservoir was used in 10%. Channel stenosis was found in 13.3% of patients, with no difference noted among the techniques used. Reintervention for channel incontinence was necessary in 10% of patients, with a higher rate observed in the tapered ileal segment group (18).

Galansky et al. compared channel complication rates in children undergoing open and robotic approaches. Bladder augmentation was associated with Monti tubes or appendicovesicostomy in 34 out of 69 patients. No difference was observed between the open and the robotic groups: suprafascial channel stenosis occurred in 21.7% of patients, subfascial stenosis occurred in 7.2%, and channel incontinence was reported in 8.7% (19). Reuvers et al. reported lower channel complication rates in the pediatric population who underwent appendicovesicostomy: suprafascial stenosis occurred in 14.8% of 128 patients, with 69.5% of them having associated BA. Subfascial stenosis was less frequent, observed in 9.4% of the patients. The rate of stomal incontinence was low, affecting 6.3% of the patients (20). To reduce the leakage rate of the conduit, Macedo et al. proposed a neosphincter made from the rectus abdominal muscle (Yachia principle), with a 100% success rate in primary cases (21, 22).

Our preference for the stoma site is the umbilical site. Although the reasons are not evidence-based, patients prefer this site for its cosmetic aspect and the fact that it is invisible from the outside (as in our protocol, the patients get to meet other patients with a Mitrofanoff procedure before their own surgery). Moreover, no difference in complications rates between the umbilical site and the right lower abdominal quadrant has been found in the literature (23).

Stone formation after bladder augmentation in children is a common complication. The preventive measures mainly involve intermittent bladder flushing (24, 25). In our series, four patients (7.7%) developed stones. Two patients with bladder exstrophy reported stones: the first patient developed multiple stones 15 months after the bladder augmentation procedure and the second patient developed the stone 11 years after bladder augmentation. In the third patient, there was an ileal reservoir, and stones recurred after 4 years. The fourth patient had cloacal exstrophy and developed a bladder stone 4 years after bladder augmentation, which was treated by cystolithotomy. All four patients were not following strictly daily bladder wash to drain most of the mucus. The rate of stone complications in our series remains low compared with 13% in published series (25–27). Noordhoff et al. studied the long-term follow-up outcomes of bladder outlet procedures in 60 children with a neurogenic bladder and found a higher bladder stone rate of 38.3% (23/60), with 20 out of 23 patients having associated BA (27). To improve the quality of bladder emptying of mucus, we always use a Ch12 catheter, and after 2 years, we attempt to upgrade to a Ch14 caliber. Although we insisted on daily bladder washes after the last CIC procedure, families did not strictly follow the advice. In van den Heijkant et al.’s series, this regular flushing on 28 ileocystoplasties helped minimize the rate of stone formation, with no UTI reported after 48 months of follow-up (24). Although infection is a common factor in stone development (28), none of our patients who developed bladder stones had previous UTI. Mucus accumulation was most likely the main factor in our population.

## Conclusion

The serous-lined continent outlet associated with W-shaped ileal bladder augmentation is a durable and efficient technique for treating children with incontinence caused by different etiologies. Compared with other published techniques, our study reported a relatively low complication rate, specifically for channel strictures, in this complex group of patients.

## Data Availability

The raw data supporting the conclusions of this article will be made available by the authors without undue reservation.
